# 18-FDG PET/CT assessment of basal cell carcinoma with vismodegib

**DOI:** 10.1002/cam4.33

**Published:** 2012-09-17

**Authors:** Curtis A Thacker, Glen J Weiss, Raoul Tibes, Lisa Blaydorn, Molly Downhour, Erica White, Jason Baldwin, Daniel D Hoff, Ronald L Korn

**Affiliations:** 1 Scottsdale Medical Imaging, Ltd.Scottsdale, AZ; 2 Midwestern UniversityGlendale, AZ; 3 Virginia G. Piper Cancer Center Clinical Trials at Scottsdale Healthcare/TGenScottsdale, AZ; 4 Mayo ClinicScottsdale, AZ

**Keywords:** Basal cell carcinoma, imaging, PET/CT, vismodegib

## Abstract

The use of 18-fluorodeoxyglucose (FDG) positron emission tomography with computed tomography (PET/CT) in subjects with advanced basal cell carcinoma (BCC) has not been fully explored due to the rarity of disease presentation. This study evaluated PET/CTs from subjects with advanced BCC participating in a phase I dose-escalation clinical trial of vismodegib. Fourteen subjects with BCC were imaged with 18-FDG PET/CT for lesion identification and response categorizing (European Organisation for Research and Treatment for Cancer [EORTC] and PET response criteria in solid tumors [PERCIST] 1.0). Several parameters including metabolic activity of target lesions, site of disease presentation and spread, treatment response, and prognostic significance of metabolic activity following therapy were evaluated. All subjects exhibited at least one hypermetabolic lesion. Most subjects had only four organ systems involved at study enrollment: skin–muscle (93%), lung (57%), lymph nodes (29%), and bone (21%). SUVmax measured across all lesions decreased (median 33%, SD ± 45%) following therapy with metabolic activity normalizing or disappearing in 42% of lesions. No significant difference was observed between EORTC and PERCIST 1.0. Subjects that demonstrated at least a 33% reduction in SUVmax from baseline had a significantly longer progression-free survival (PFS) (median 17 months, 95% confidence interval [CI] ±4 months vs. 9 months, 95% CI ±5 months, *P* = 0.038) and overall survival (OS) (median 24 months, 95% CI ±4 months vs. 17 months, 95% CI ±13 months, *P* = 0.019). BCC lesions are hypermetabolic on 18-FDG PET/CT. A decrease in SUVmax was associated with improved PFS and OS. These results further support the incorporation of 18-FDG PET/CT scans in advanced BCC management.

## Introduction

Approximately 1.4 million cases of cutaneous basal cell carcinoma (BCC) are diagnosed every year in the United States, making BCC the most common form of malignant skin cancer [1]. A rising incidence has been reported since the 1960s, increasing by up to 2% per year in some geographic locations [2]. BCC lesions can occur anywhere on the body, but most commonly they occur on skin that routinely receives sun exposure, such as the head and neck. The vast majority of BCC lesions are locally controlled by surgery. However, although rare, BCC can metastasize at an incident rate of only 0.0028–0.1% [3, 4]. Recent molecular studies have highlighted a mutation in the Hedgehog pathway genes (patched homolog 1 [PTCH1]) that can lead to uninhibited cutaneous basal cell proliferation via activation of smoothened homolog (SMO) a transmembrane signaling protein [5, 6]. Both are components of the Hedgehog-signaling pathway (HHSP), and the impetus for recent pharmaceutical intervention using targeted therapy to SMO [7, 8]. SMO antagonists could provide an alternative to surgical excision or a palliative therapeutic option for locally advanced and metastatic BCC. Recently, the FDA approved the use of vismodegib (a SMO antagonist) in treatment of advanced BCC.

One of the challenges in BCC is the lack of standardized imaging methodologies to evaluate BCC lesions, as these tumors are often times poorly defined and have an infiltrative appearance on conventional imaging techniques such as computed tomography (CT) and magnetic resonance imaging (MRI) (i.e., evaluable with these methodologies but not measurable). The anatomic and physiologic data obtained by 18-fluorodeoxyglucose (FDG) positron emission tomography with CT (PET/CT) have proven to be an integral part in the management of patients with many different types of malignancies [9–11]. In addition, PET/CT might be of benefit in the advanced disease population due to whole-body imaging with PET/CT and a more favorable analysis of functional rather than anatomic properties. Historically, however, 18-FDG PET/CT has not been used to assess BCC due to the rarity of advanced disease, metastatic presentation, and lack of sufficient clinical information regarding the utility of 18-FDG PET/CT in this patient population. Assessing response of tumors to targeted biologically active agents is becoming increasingly important and 18-FDG PET/CT can provide clinicians the ability to make more informed treatment decisions.

In this study, we explored the value of 18-FDG PET/CT in patients with advanced BCC who were treated with vismodegib as part of a phase I clinical trial [8]. In addition, 18-FDG PET/CT was also evaluated for utility in determining treatment response.

## Patients and Methods

### Patient selection

Written informed consent was obtained from all patients who participated in the clinical trial with vismodegib (http://ClinicalTrials.gov number, NCT00607724) [8]. Our local institutional review board approved this retrospective radiographic study. This report reviews the 14 subjects who were part of the clinical trial at a single site. All 14 subjects had baseline (pre-SMO antagonist treatment) and successive 18-FDG PET/CT scans at approximately 60-day intervals, until disease progression, dose-limited toxicity, or death. There were 10 men and four women. The average age was 66 years for men and 50 years for women. All patients enrolled on this study had staging of their disease. For a patient to be categorized as metastatic (stage IV) BCC, a biopsy of a metastatic lesion was previously confirmed by histopathologic review. Six subjects had American Joint Committee on Cancer stage III disease and eight subjects had stage IV disease. Prior to enrollment, 9 (64%) of 14 had not received prior systemic chemotherapy, while 5 (36%) of 14 received prior systemic chemotherapy, including two subjects with stage III and three subjects with stage IV disease.

### PET/CT imaging

Before imaging, patients were instructed to fast for at least 4 h. Fasting blood glucose was measured before IV injection of 18F-FDG; patients meeting the criterion of a blood glucose level within the 60–200 mg/dL range received 18F-FDG (0.154 mCi/kg or 0.57 mBq/kg) through an antecubital vein. After injection, patients were instructed to remain in a recumbent position for 1 h (or 1.5 h for body mass index [BMI] >30) in a quiet environment. After having voided, each patient was placed supine on the PET/CT scanner (Discovery STE, GE Healthcare, Waukesha, Wisconsin); the field of view was 70 cm, and the full width at half maximum (FWHM) was 6.5 mm.

The CT attenuation data were acquired at 140 kVp, 80 mAs, a pitch of 1.5:1, a table speed of 15 mm/rotation, and 5-mm slice thickness in a craniocaudal direction while the patient was undergoing tidal breathing. PET emission data were then acquired in a caudocranial direction progressing from the thighs to the orbitomeatal–vertex line at 3–5 min per table position. The PET/CT data sets were reconstructed using iterative reconstruction (ordered subset expectation maximum [OSEM]) with attenuation correction applied. The images were displayed on a workstation (Advantage Workstation v4.3, GE Healthcare) in the axial, coronal, and sagittal planes.

### Quantification of lesion activity

Standardized uptake value (SUV) calculations were obtained by placing a user-defined circular region of interest (ROI) on the attenuation-corrected images resulting in a display of the mean and maximum SUVs (SUVmean and SUVmax, respectively). SUV is defined as the amount of 18F-FDG activity in an ROI per gram of tissue according to the following formula: SUV = activity/mL tissue (decay corrected)/injected dose/body weight. All PET/CT studies were interpreted by an expert nuclear medicine radiologist with >20 years of experience in the interpretation of PET scans (R. L. K.).

### Imaging and lesion analysis

Independent analysis of 18-FDG PET/CT scan for each subject was performed by retrieving images from a picture archiving and communication system (PACS) and analyzing each scan on a GE Advantage Workstation volume sharing version 4.3. A SUVmax value of target lesions (lesions measuring at least 1 cm and with visual activity above organ background according to the criteria of European Organisation for Research and Treatment for Cancer [EORTC] and PET Response Criteria in Solid Tumors PET response criteria in solid tumors [PERCIST], version 1.0) was selected [12, 13]. A ROI was deposited on the image slice with the hottest activity. No more than a total of five lesions per subject were chosen as target lesions. The SUVmean of liver and mediastinum blood pool on image slices that were free of disease was obtained to assess systemic metabolic uptake for each 18-FDG PET/CT scan for the PERCIST criteria. At the initial 18-FDG PET/CT scan, subject disease burden was estimated by using the number of metabolically active lesions present on the whole-body 18-FDG PET/CT scan above normal background organ activity and separating them into one of the following categories: 1 lesion, 2–5 lesions, 6–25 lesions, and >25 lesions. SUV values were recorded. These lesions and any new (postbaseline) lesions were then evaluated using the same criteria applied to each subsequent PET/CT scan.

### Response analysis

The EORTC and PERCIST 1.0 criteria [12, 13] defined and summarized in Table 1. The best response was defined as the greatest percentage decrease in sum of total SUVmax compared with the total sum at baseline scans. Progression-free survival (PFS) (calculated as the time from start of SMO to disease progression or data cutoff) and overall survival (calculated as time from start of SMO to time of death or data cutoff) (OS) were recorded. Additionally, RECIST 1.0 criteria using physical examination and/or CT imaging were recorded [12].

**Table 1:** EORTC and PERCIST 1.0 criteria

Category	EORTC	PERCIST 1.0
Complete metabolic response (CMR)	100% reduction in FDG avidity of target lesion and no new lesions	100% reduction in FDG avidity of target lesion and no new lesions
Progressive metabolic response (PMR)	15% reduction in SUV after first cycle and 25% reduction in SUV after second cycle and no new lesions	30% reduction in SUV and reduction of at least 0.8 in absolute value; no new lesions
Progressive metabolic disease (PMD)	>25% increase in SUV from baseline and or new lesions	>30% increase and >0.8 increase in SUV from baseline; new lesions
Stable disease (SD)	SUV between PMR and PMD	SUV between PMR and PMD
Duration of response	Not determined	CMR to date of PMD or CMR/PMR to date of PMD or SD from date noted to date of PMD

Adapted from references [12, 13].

### Statistical analysis

All data were retained in Microsoft Excel 2008 version 12.0. Statistical methods of all collected values were performed using Student's *t*-test. Kaplan–Meier plots were created using MedCalc Software program (Ver. 11.6.0, 2011). Significance was considered if the *P*-values <0.05.

## Results

### BCC lesion activity

All 14 subjects exhibited at least one hypermetabolic lesion on baseline scans. A total of 50 hypermetabolic lesions were chosen as target lesions and followed on subsequent scans. On average, each subject underwent six 18-FDG PET/CT scans (range 2–15). Pre-SMO antagonist treatment lesion analysis demonstrated an average SUVmax of 7.6 (median SUVmax, 6.5; standard deviation [SD] 4.6, 95% confidence interval [CI] ±1.3) per target lesion.

### Distribution of FDG avid BCC lesions

**Figure 1:**
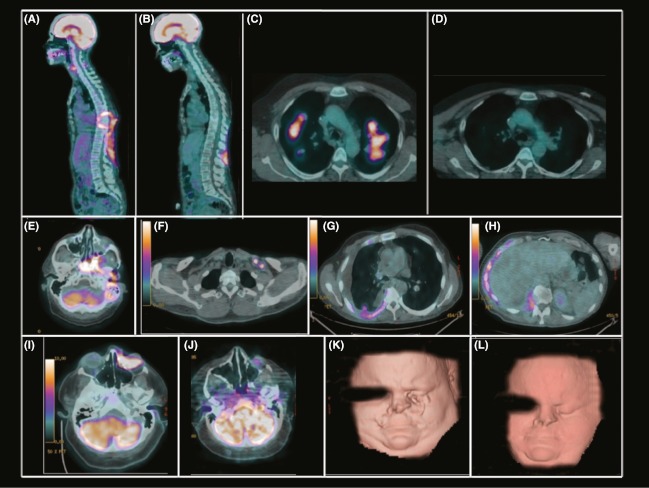
Representative images from 18-FDG PET/CT scans. Panels A–D represent baseline and follow-up 18F-FDG PET/CT scans in two separate subjects with soft tissue, bone, and muscle involvement (panels A and B; SUVmax 5.6 vs. 2.8 baseline vs. follow-up, respectively) and lung involvement (panels C and D; SUVmax 12.6 vs. 0.2 at baseline vs. follow-up, respectively). Panels E–H are sample images demonstrating typical locations of BCC metastasis on 18-FDG PET/CT in the skull base (E), lymph nodes (F), lungs, bone, and pleural space (G and H). Panels I–J show the value of baseline axial 18-FDG PET/CT image and three-dimensional CT volume rendering depicting the extent of superficial and deep tissue lesion involvement of a hypermetabolic lesion (SUVmax 21.1; panels I and K) of the inferior left orbit, left cheek, nasal tissues, and maxillary tissue. After therapy, the axial 18-FDG PET/CT fusion image and three-dimensional CT volume rendering exhibited significant reduction in metabolic activity (SUVmax <2.0) and soft-tissue healing (panels J and L).

At the time of study analysis, only four organ systems were affected in metastatic spread of the tumor, namely skin–muscle (13/14, ∼93%), lung (8/14, ∼57%), lymph nodes (4/14, ∼29%), and bone (3/14, ∼21%) (Fig. 1). Disease burden was low (only one lesion) in three of 14 subjects compared with four of 14 subjects that had a moderate (2–5 lesion) disease burden. The highest disease burden (6–25 lesions) was observed in seven of 14 subjects. Following therapy, lesions in the lung and skin–muscle demonstrated the highest decrease in SUV of 56% (*P* < 0.01) and 49% (*P* < 0.01), respectively, whereas bone lesions exhibited the lowest decrease in maximum SUV of 16% (*P* = 0.29) (Table 2).

**Table 2:** Lesions, metastasis, SUV, and response in BCC

Subject (age, gender)	Estimated disease burden (total number of lesions)	Target lesion mean SUVmax (min–max)	EORTC best response (% change following treatment)	RECIST best response	PFS (days)	OS (days)	Location of primary lesion(s)	Location of metastatic lesion(s)	Reason for progression
1 (49, M)	6–25	9.2 (3.7–15.3)	CMR (−100%)	PR	606	606 (C)	Head and neck	Lung	C
2 (59, M)	6–25	14.8 (10.3–21.7)	SD (−3%)	PD	63	131	Head	Lung and bone	P
3 (84, M)	6–25	6.8 (5.1–8.5)	SD (−21%)	PR	262	843	Head and neck	Lung	P
4 (67, M)	6–25	8.7 (7.1–10.1)	PMR (−70%)	PR	566	860	Head and neck	Lung and lymph nodes	P
5 (49, M)	6–25	10.1 (7.4–12.6)	PMR (−75%)	PR	539	585 (C)	Neck	Lung	P
6 (57, M)	6–25	7.7 (4.1–11.7)	SD (−48%)	SD	120	698	Back	Lung and bone	N
7 (65, M)	6–25	5.3 (3–10.1)	PMR (−71%)	SD	319	546	Back	Lung and lymph nodes	P
8 (62, F)	6–25	3.9 (2.8–5.6)	PMR (−41%)	PR	314	602 (C)	Back	Lymph node	P
9 (86, M)	1	13.1	PMR (−92%)	SD	617	1035 (C)	Head and neck	Skin and muscle	N
10 (50, F)	1	5.6	SD (−18%)	SD	460	594	Head	Skull and globe	P
11 (41, F)	2–5	10.8 (3.8–21.1)	PMR (−82%)	SD	538	538 (C)	Head	Lymph node and bone	P
12 (49, M)	2–5	5.6 (4.8–6.3)	PMR (−63%)	PR	291	964 (C)	Back	Skin and muscle	P
13 (48, F)	2–5	5.0 (2.8–6.6)	PMR (−57%)	PR	231	977 (C)	Chest	Lung	N
14 (61, M)	1	5.4	PMR (−50%)	SD	289	642 (C)	Chest	Bone	N

M, male; F, female; SUV, standardized uptake value; PFS, progression-free survival; OS, overall survival; C, censored; N, new; P, progression of metabolic activity on 18-FDG PET/CT.

### Response criteria measurements

By RECIST 1.0 measurements (physical examination and/or imaging), there were seven partial responses, six stable disease, and one progressive disease as the best response. The best response by both EORTC and PRECIST 1.0 criteria was one CMR, nine PMR, and four SD. Comparing RECIST 1.0 with EORTC/PRECIST 1.0 categorizations, there were six discordances (the PR and CMR for Patient #1 is considered concordant) (not significant by Fisher's exact test). The patients with discordances for EORTC versus RECIST are Patients 2, 3, 7, 9, 11, and 14 (Table 2).

The median metabolic duration of response in days was 168 (SD 189.4, 95% CI ±111.9). The SUVmax measured across all lesions decreased by an average of 25% (median 33%, range: 100% decrease to 74% increase). The metabolic activity normalized or disappeared in a total of 21 of 50 target lesions (42%). Disease progression based on the development of a new lesion(s) was noted in four of 14 patients (Table 2). There was no observed difference between when a response was detected and disease burden. There was also no observed difference between disease burden and degree of response. No consistent pattern in the timing of response relative to the initiation of therapy or the extent of SUVmax change was observed.

### Prognostic information of PET/CT scans

**Figure 2:**
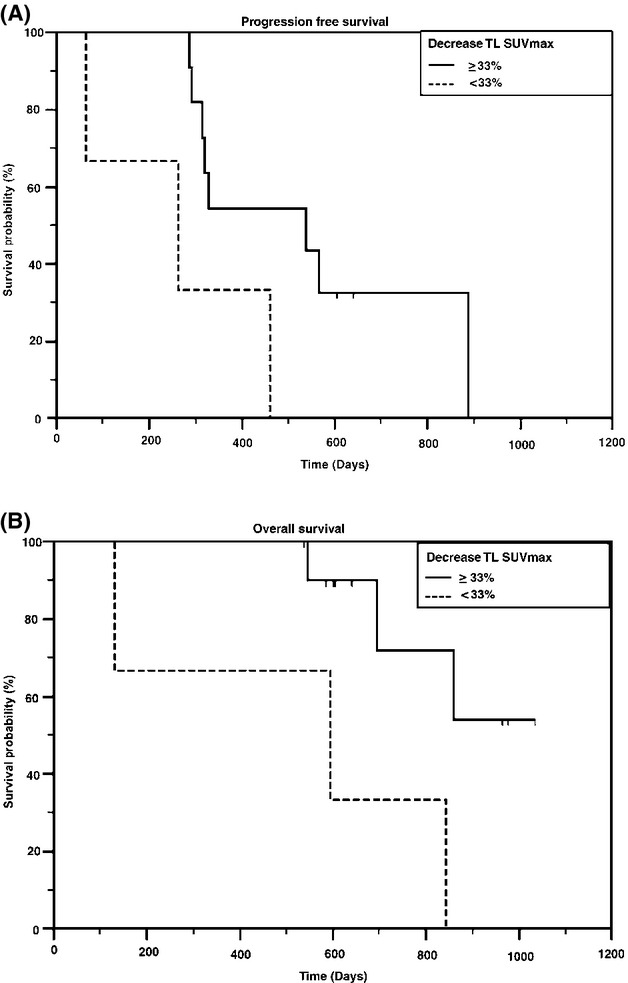
Progression-free survival (*P* = 0.038) (A) and overall survival (*P* = 0.019) (B); Kaplan–Meier (censored) curves demonstrating significant differences in days between subjects with at least 33% reduction (solid line) in SUVmax compared to those exhibiting less than 33% reduction in SUVmax (broken line).

In order to determine the association between the prognostic value of the best 18-FDG PET/CT response and the clinical endpoints of PFS and/or OS, an empiric approach was used to determine the lowest SUVmax change that would be needed to separate out subjects with significant differences in PFS and OS. Subjects with at least a ≥33% reduction (*n* = 10) in total SUVmax from baseline had a significantly longer PFS (*P* = 0.028; median 502.9 vs. 269 days) and OS (*P* = 0.019; median 708.6 versus 522.6 days; Fig. 2). Most of these subjects (7/10) achieved at least a 33% reduction in metabolic activity after the first PET/CT scan around day 60, while 3 of 10 of subjects had a 33% or greater reduction in total SUVmax around 171 days after treatment, that is, between their second and third PET/CT scan. No PET/CT scans were performed before 50 days.

## Discussion

The clinical management of patients with different types of malignancies often involves PET/CT to evaluate overall tumor burden and disease response. There is also growing evidence that the decline in FDG tumor metabolism after therapy contains important prognostic information [14]. However, not all malignancies, including BCC, have been thoroughly evaluated by 18-FDG PET/CT. As most patients with BCC are treated with surgery and do not present with advanced disease, there has been little opportunity until now to be able to use of PET/CT in BCC. Much of the opportunity to apply PET/CT in this study has been driven by a phase I clinical trial using targeted therapy with a SMO antagonist [8]. Hypermetabolic primary BCC lesions have previously been reported on the head and neck [15] using PET, but to our knowledge, this is one of the first series to report the experience of PET/CT in the assessment of treatment response for advance stage BCC.

BCC lesions are hypermetabolic on 18-FDG PET/CT. The average SUVmax per lesion noted in this study is 7.6. The distribution of BCC lesions in this report revealed that not all organ sites were found to contain metastatic lesions. Only skin–muscle, bone, lung, and lymph nodes were noted to have involvement on PET/CT. Interestingly, no metastatic lesions were observed in the other organ systems in this population (e.g., spleen, pancreas, adrenal glands, intestines, or brain). The extremely high metastatic occurrence in such a limited number of organ sites may reflect a unique tumor biology of BCC, host environment, or both, or the relatively small sample size of our population.

The significant reduction in FDG activity with targeted therapy noted here was in keeping a biological response. Lesions in the lung and skin–muscle demonstrated on average a higher reduction in SUVmax (49–56%) compared with those in bone and lymph nodes (16–30%). One possible reason could be that subtype differences in BCC (original pathology was not available in all patients) can affect FDG uptake [16], although there is debate to the extent of metastatic potential of various BCC histological subtypes [17]. Differences in tissue perfusion and local environment could impact the lesion site, but this has not yet been established. Given the small number of subjects, this observation needs to be confirmed in larger patient cohorts.

Deriving prognostic data from 18-FDG PET/CT is currently thought to reside in effective categorization of patient response to treatment. Different methods [12, 13] (EORTC, PERCIST, etc.) have been proposed, but no single method has been fully accepted due to inconsistencies of predicted outcomes between different malignancies. In this study, there was no difference between EORTC versus PERCIST 1.0 categorization. There was a significant linkage between loss of FDG metabolism and clinical endpoints of PFS and OS. In particular, the results in this article demonstrated that at least a 33% reduction in total SUVmax during treatment was correlated with a longer PFS and OS compared with those who did not achieve a 33% reduction. These results suggest that the metabolic changes observed with 18-FDG PET/CT in patients with BCC treated with a unique-targeted therapy may be correlated with clinical response by using this empiric threshold of SUVmax reduction from the baseline scan. Such correlations have been found in other types of malignancies. For example, changes in metabolic activity observed with 18-FDG PET/CT have been used to predict overall response in patients with different malignancies like Hodgkin's lymphoma and metastatic pancreas cancer [18, 19]. It appears from our data that 18-FDG PET/CT response was more informative than RECIST criteria and 42.3% had discordances for best response. A large phase II trial was reported on patients treated with vismodegib in advanced BCC, and there is an ongoing phase II trial using LDE225, another SMO antagonist, in advanced BCC [20, 21]. As clinical experience with treating advanced BCC with SMO antagonist increases, the relationship can be further defined.

Although there was no consistent pattern in timing of best response relative to the initiation of therapy or extent of SUVmax change, the average time to reach a best 18-FDG PET/CT response was 183 days (median 148 days ± 62 days 95% CI). The mean progression-free survival was 436 days (median 395 days ± 109 days 95% CI). These results suggest that best response is seen within the first half of the PFS interval.

One of the major limitations of this study was its small sample size. As noted before, advanced and metastatic BCC is a rare presentation of a common skin malignancy. Thus, most studies with modest to large subject populations with advanced, metastatic BCC will be difficult to accrue. Nevertheless, this study demonstrates the potential value of 18F-FDG PET/CT in this patient population for determining the extent of disease involvement and treatment response.

BCC, the most common malignancy in the world, is paradoxically the least described, evaluated, or managed with 18-FDG PET/CT. This study demonstrates that BCC lesions are hypermetabolic on 18-FDG PET/CT. BCC metastases were not evenly distributed to all organ systems, but found in high frequencies in skin–muscle, bone, lymph nodes, and lung. Unlike RECIST 1.0 categorization, there was no difference in response categorization using either EORTC or PERCIST 1.0 criteria. There was a significant correlation between 18-FDG PET/CT response and PFS and OS. These results point to the valuable role that 18-FDG PET/CT might have in the management of patients with advanced BCC.
